# Successional Dynamics in the Gut Microbiome Determine the Success of *Clostridium difficile* Infection in Adult Pig Models

**DOI:** 10.3389/fcimb.2019.00271

**Published:** 2019-08-06

**Authors:** Stephanie D. Jurburg, Jan J. B. W. J. Cornelissen, Paulo de Boer, Mari A. Smits, Johanna M. J. Rebel

**Affiliations:** ^1^Wageningen Bioveterinary Research, Lelystad, Netherlands; ^2^iDiv – German Centre for Integrative Biodiversity Research, Leipzig, Germany; ^3^TNO Innovation for Life, Zeist, Netherlands; ^4^Wageningen Livestock Research, Wageningen, Netherlands

**Keywords:** *Clostridium difficile*, pig, microbiome, animal models, bacteria

## Abstract

*Clostridium difficile* infections (CDI) are a major cause of antibiotic-associated diarrhea. It is hypothesized that CDI develops due to the antibiotic-induced disruption of the intestinal microbial community structure, which allows *C. difficile* to flourish. Here, we pre-treated weaned pigs with the antibiotics Clindamycin or Ciprofloxacin for 1 day, and subsequently inoculated them with a human and pig enteropathogenic *C. difficile* strain 078 spores. Body temperature, clinical signs of disease, and the fecal microbiome were monitored daily for 15 days. Clindamycin had a stronger effect on the pigs than Ciprofloxacin, resulting in drastic shifts in the fecal microbiome, decreases in microbial diversity and significant increases in body temperature, even in the absence of *C. difficile*. Fecal shedding of *C. difficile* was detectable for 3 and 9 days in Ciprofloxacin and Clindamycin treated pigs inoculated with *C. difficile*, respectively, and in both cases decreased cell proliferation rates were detected in colon tissue. The timing of *C. difficile* shedding coincided with the decrease in a large cluster of Firmicutes following Clindamycin treatment, a pattern which was also independent of *C. difficile* inoculation. The observed community patterns suggest that successional dynamics following antibiotic treatment facilitate *C. difficile* establishment. The similarities between the microbiome responses observed in our study and those previously reported in CDI-infected humans further support the utility of adult pigs as models for the study of CDI.

## Introduction

*Clostridium difficile* infections (CDI) are a major cause of hospital-associated and antibiotic-associated diarrhea in humans (Monaghan, 2015). The clinical manifestations of CDI vary and range from mild diarrheal symptoms to severe colitis. Complications associated with CDI include bowel perforation, renal failure, sepsis, and systemic inflammatory response syndrome. CDI patients can be treated with specific antibiotics, although 20% encounter a recurrent episode (Laffan et al., 2006; Marsh et al., 2012). It is hypothesized that the residing gut microbiota is important in the maintenance of *C. difficile* colonization resistance in healthy individuals, and that CDI is associated with dysbiosis of the intestinal microbiota caused by antibiotic use (Reeves et al., 2011). Antibiotics disrupt the intestinal microbial community structure, decreasing its colonization resistance and promoting the germination and colonization of *C. difficile* spores in the colon (Paterson, 2004). The shift in microbial community structure that induces sensitivity to *C. difficile* infections has not been studied at a high temporal resolution due to lack of longitudinal studies which sample the intestinal microbiome prior to antibiotic administration and *C. difficile* infection.

The understanding of how colonization mechanisms by *C. difficile* precludes the development of therapeutic and preventive methods to treat infections (Kociolek and Gerding, 2016). Fecal microbiota transplantation is an established treatment for recurrent CDI, as it restores microbial homeostasis and diversity (Choi and Cho, 2016). However, there are numerous long-term safety and regulatory issues associated with this practice. Novel preventive prophylactic therapies, which increase the native microbiota's resistance to CDI are in diverse states of development (Best et al., 2012). Nutrition-based treatments may contribute toward increasing the native intestinal microbiota's resistance to colonization by *C. difficile*, but to date are underexplored, largely because the diets and digestive systems of current murine models differ from the human digestive system (Best et al., 2012; Schubert et al., 2015).

In order to rigorously examine the potential success of various treatments, it is crucial to have an animal model with a comparable digestive system and diet to humans. An alternative to murine models are pig (*Sus scrofa domesticus*) models. Recent literature indicates that pigs are a valuable translational model in developing therapies for human health, especially nutritional studies, intestinal microbiota associated diseases, and immune parameters (Litten-Brown et al., 2010; Meurens et al., 2012; Heinritz et al., 2013). The post-weaning gut development and nutritional requirements of pigs closely resemble those of humans (Andersen et al., 2011; Heinritz et al., 2013). After weaning, pigs exhibit similar physiology, morphology, and functionality of the gastro-intestinal tract (GIT) to humans, and microbial and immune homeostasis have already been established. Additionally, CDI has been documented to cause enteric disease in pigs (Goorhuis et al., 2008; Debast et al., 2009). In one study, the effects of CDI in germfree piglets and 1–10 day-old non-weaned conventional piglets included short periods of diarrhea in some piglets, while others were non-diarrheic but showed colitis at necropsy (Arruda et al., 2013).

Since the relationship between gastrointestinal microbial dysbiosis and CDI development has frequently been observed, it is hypothesized that specific microbial community structures may be indicative of an increased risk of CDI (Britton and Young, 2014). Studying the relationship between an individual's native gastrointestinal microbiome CDI requires the collection of longitudinal fecal samples before and immediately after the onset of CDI, which is not possible in humans. The objectives of this study were (1) to explore the dynamics of the gastrointestinal microbiome before and during CDI following antibiotic administration, using 8–10 week old pigs, and (2) to establish adult pigs as models for the study of CDI. To induce microbial dysbiosis and simulate the development of CDI in humans, we administered the pigs with Clindamycin or Ciprofloxacin, both of which are associated with increased risk for CDI in humans. Clindamycin has become the inducing agent of choice in small animal models (Best et al., 2012), while most *C. difficile* strains have been found to be resistant to Ciprofloxacin (Norman et al., 2014). Both antibiotics are commonly used to treat bacterial infections in humans. Following antibiotic administration, we challenged the pigs with *C. difficile*, and monitored various health parameters, as well as their fecal microbiome for the following 15 days. By monitoring the microbiome before and after antibiotic administration and *C. difficile* inoculation, we aim to provide further insight into the relationship between the native gut microbiome, the effect of antibiotic treatment, and susceptibility to CDI, and to establish the pig gut as a suitable model for the study of CDI in humans.

## Materials and Methods

### Experimental Design

All procedures were approved by the animal experimentation board at Wageningen University & Research Center (accession number AVD401002015141) and carried out according to the guidelines of the European Council Directive 86/609/EEC dated November, 1986. Briefly, 25 seven week-old pigs were obtained from a commercial breeder 2 weeks after weaning. The pigs were divided into five groups of five animals each, and housed in five separate pens. Prior to the beginning of the experiment, the pigs were allowed to adapt for 1 week (Figure S1). All pigs were fed the same controlled feed. At day −1 of the experiment, two groups (Clin *C. diff* and Clin) were orally administered with 50 mg/kg Clindamycin (TEVA Nederland BV, Haarlem, the Netherlands); two groups (Cip *C.diff* and Cip) with 15 mg/kg Ciprofloxacin (Pharmachemie B.V., Haarlem, the Netherlands); and one group served as a control group. At day 0 and 16 h later, the pigs in groups Clin *C. diff* and Cip *C.diff* were intra-gastrically inoculated with 2 × 10^9^ CFUs of *C. difficile* ribotype 078. Groups Clin and Cip were intra-gastrically inoculated with sterile water. Feces were collected on days −5, 0, and daily between days 1 and 15 by taking rectal fecal samples. Blood was collected and body weights were measured on days −5, 0, 5, 10, and 15. Body temperature was measured once a day, starting on the day of arrival and twice daily after day 1 (Figure S1). During the experimental phase pigs were also monitored for clinical signs of disease, including diarrhea, dehydration, dyspnoea, weakness, lethargy, and anorexia. At the end of the experiment, animals were anesthetized with intravenous 20% 1 ml euthasol (AST Farma B.V. Oudewater, The Netherlands) and euthanized to collect tissue samples.

### Preparation of the *C. difficile* Inoculum

A toxigenic *C. difficile* strain of PCR Ribotype 078 (human isolate, kindly given by Prof. E. Kuijper, Centre for Infectious Diseases, Leiden University Medical Centre, Leiden, The Netherlands) was selected for the experimental *C. difficile* challenge because this strain has been found in both humans and in pigs (Goorhuis et al., 2008). Spores of *C. difficile* Ribotype 078 were prepared as follows. *C. difficile* was anaerobically grown in Schaedler Anaerobe broth (SAB, Oxoid CM0497) for 5 to 7 days at 37°C. Spores were harvested by centrifugation and washed with cold water at least three times. *C. difficile* spores were selected by heat treatment for 20 min at 65°C to ensure that any remaining vegetative cells were killed. Viable spores were calculated to determine the *C. difficile* inoculation dose by plating for colony forming units (CFU/ml). *C. difficile* strain 078 was shown to be vulnerable to Clindamycin (TEVA Nederland BV, Haarlem, The Netherlands) and Ciprofloxacin (Pharmachemie B.V., Haarlem, The Netherlands), allowing the use of both antibiotics in the subsequent experiments.

### Histopathology and Immunohistochemistry

Histopathology was performed at the end of the experiment to detect CDI-related changes in the intestines of all pigs. Formalin-fixed colon and jejunum tissue sections (5 μm thick) were stained with haematoxylin and eosin (HE staining) and were examined morphologically by light microscopy for changes in the crypt depth and villus height as previously described (Zekarias et al., 2005).

Immunohistochemistry was performed to identify CDI-related changes in the colon and jejunum of pigs from all other treatments. Frozen colon and jejunum tissue sections (10 μm thick) were immunohistochemically stained for the detection of CD3+ T-cells (abcam ab16669) and Macophages (CVI-SwNL 517.2 ID nr: 107) by an indirect immunoperoxidase method as described before (Bianchi et al., 1992). The images were acquired and analyzed with Image-Pro Plus (version 5.1, DVC Machinevision Breda, The Netherlands). Proliferating cell nuclear antigen (PCNA) was used as an indicator for cell health; a decreased PCNA may indicate reduced cell renewal rates and intestinal lesions. PCNA was detected by immunohistochemistry in formalin-fixed duodenum and jejunum tissue sections (rat PCNA; PC-10, Dako, Glostrup, Denmark) in PBS containing 0.1% BSA. The cellular markers were visualized using Dako Real TM EnvisionTM Detection System-HRP (K5007ENV) according to the manufacturer's instructions. Examination of the slides under light microscopy and scoring were as described before (Zekarias et al., 2005). All animals were examined, and the mean scoring was calculated for the group at a given time period.

### DNA Isolation and Quantification of Bacterial Community and *C. difficile* in Fecal Samples

DNA was isolated from snap-frozen fecal samples according to Ladirat et al. (2013) and used for quantitative PCR (qPCR) analysis as well as sequencing. The amount of *C. difficile* DNA was determined by quantitative qPCR. qPCR was performed on an ABI 7500 Fast System (Taqman-MGB) using in-house designed primer-probe combinations targeting *C. difficile*-specific portions of the 16S rRNA gene (Cdif Forward 5′-GCAACGCGAAGAACCTTACCTA-3′; Cdif Reverse 5′- GAAGGGAACTCTCCGATTAAGGA-3′; Cdif probe 5′- TGACATCCCAATGACA-3′; VIC-MGB). A standard curve spanning seven orders of magnitude (10^0^-10^6^ fg) was generated using total chromosomal DNA of the target strain. The amount of detected target sequence was calculated as femtograms according to this standard curve. Total bacterial DNA was determined by targeting the 16S rRNA gene, using the generic PCR primer-probe combination (16S uni-I-F 5′- CGA AAG CGT GGG GAG CAA A-3′; 16S uni-I-R 5′- GTT CGT ACT CCC CAG GCG G-3′; 16Suni-I 5′- ATT AGA TAC CCT GGT AGT CCA-3′; FAM-MGB). qPCR conditions for both reactions are detailed in Section S2. *C. difficile* counts were divided by total 16S rRNA counts per sample to account for differences in fecal swab content between samples. The detection limit was set as (*mean* 16*S rRNA counts*)^−1^.

### Bacterial Community Sequencing and Processing

The V4 region of the 16S rRNA gene was amplified by PCR as described by Caporaso et al. (2012). Amplicons were confirmed by analysis on a fragment analyzer (Advanced Analytical, Germany), after which the pool was purified by the Qiaquick gel extraction kit from (QIAGEN) and sequenced by targeted-amplicon 16S sequencing on an Illumina MiSeq sequencer as previously described (Caporaso et al., 2012). All processing and analyses were performed in R 3.4.0 (R Core Team, 2014). The 16S rRNA gene sequencing reads were filtered, trimmed, dereplicated, chimera-checked, and merged using the dada2 package (Callahan et al., 2016) and reads were assigned with the RDP classifier (Wang et al., 2007). Downstream analyses were performed with the phyloseq (McMurdie and Holmes, 2013) package. Reads were rarefied to 4 686 reads per sample using the rarefy_even_depth function (seed = 1). The final dataset contained 10 604 745 reads distributed among 2 969 sequence variants (SVs). Good's coverage was >0.999.

### Statistical Analyses

Statistical analyses were performed in R 3.4.0 (R Core Team, 2014) using the vegan (Oksanen et al., 2013) and phyloseq (McMurdie and Holmes, 2013) packages. Richness, or α-diversity was calculated using the Shannon-weiner index and total observed SV richness. Differences between time and treatment for 16S rRNA qPCR counts and α-diversity were evaluated using two-way ANOVAs or one-way ANOVAs followed by *post-hoc* Tukey tests for pairwise comparisons. Differences between genera and phyla on day −5 and day 0 within treatments were evaluated with Welch's *t*-tests. Taxon abundances are displayed as *mean*± *standard deviation*.

Pairwise distances between samples were calculated using the Bray-Curtis distance metric, and visualized using a principal coordinates analysis (PCoA). Differences between treatments and time were evaluated using *adonis* on Bray-Curtis distances. Homogeneity of dispersions between replicates were evaluated using *betadisper* on Bray-Curtis distances, with Tukey tests for the evaluation of pairwise distances. A principal response curve (Van den Brink and Ter Braak, 1999) was used to compare community responses over time. We selected SVs with a species score > |1|, which indicates a strong influence on the observed community dynamics, and agglomerated them at the genus level. For the heatmap, taxa abundances were standardized per SV over time using the *decostand* function, their Euclidean distances were calculated and taxa were clustered using Ward's method. Pearson correlations between individual SVs and *C. difficile*-specific qPCR data were performed *corr.test* from the R package psych (Revelle and Revelle, 2015). The identity of a *C. difficile*-like SV was confirmed using nucleotide BLAST (Johnson et al., 2008).

## Results

### Phenotypic Observations and Pathology Associated With *C. difficile* Germination and Multiplication

**Figure 1:**
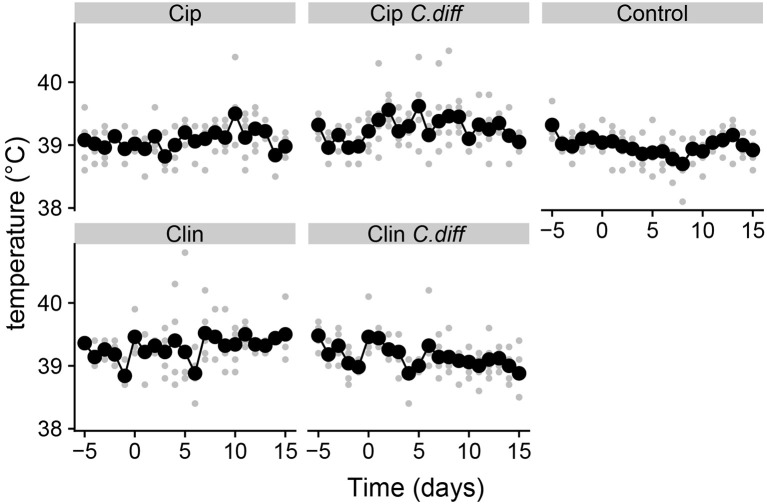
Elevated temperatures in Clindmycin and *C. difficile-*treated pigs. Rectal temperatures for pigs were significantly higher in the Clin, Clin *C. diff*, and Cip *C. diff* treatments relative to controls, on average (*p* < 0.01). A single diurnal sample is shown per pig, per day in gray. Daily means per treatment are displayed in black and connected with lines. Temperature was measured at the same time each day.

Pigs from all treatments except for the Cip group exhibited significantly higher temperatures on average compared to the control group (*P* < 0.01 for all Tukey HSD comparisons of a one-way ANOVA on the effect of treatment, Figure 1). In addition, pigs in the Cip *C. dif* group exhibited significantly higher temperatures than pigs in the Cip treatment on average (*p* < 0.0001), while pigs in the Clin treatment did not significantly differ from those in the Clin *C. diff* treatment (*p* = 0.47). The mean temperature for pigs from both the Cip *C. dif* and Clin *C. dif* treatments was highest on day 1 (39.71and 39.69°C, respectively), but neither was significantly different from temperatures exhibited before the *C. difficile* challenge (day 0).

Visual inspection of the intestine pack at necropsy showed no signs of pathological changes in any of the treatment groups. Weight gains did not differ significantly between any treatment group (data not shown).

**Figure 2:**
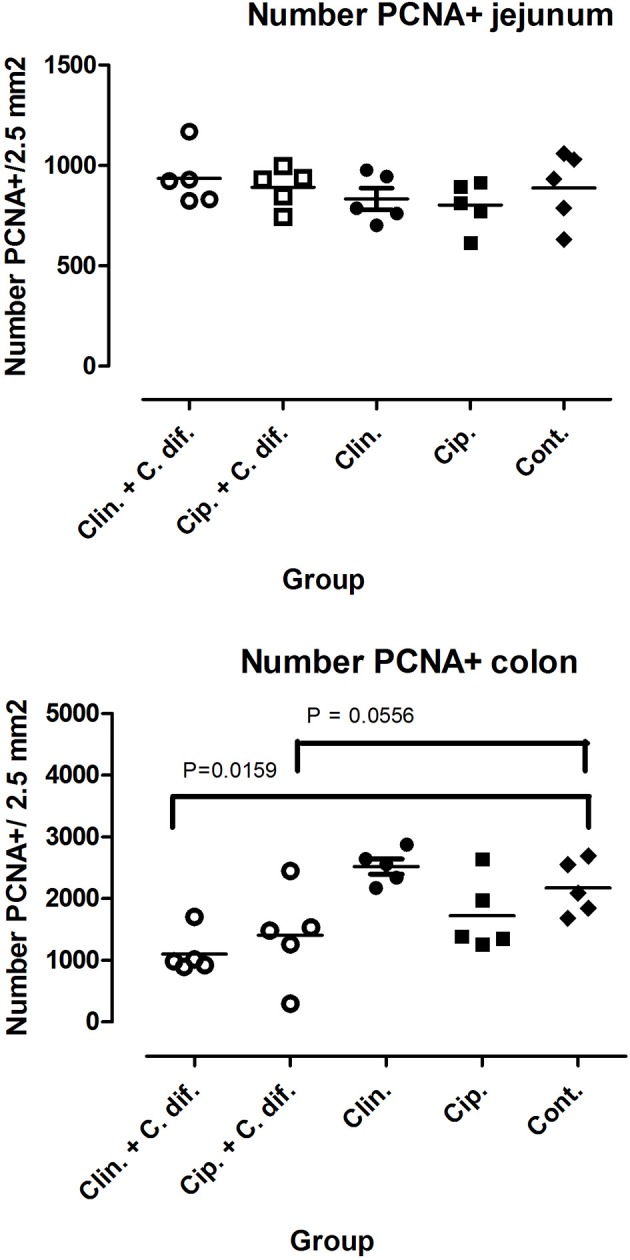
Decreased cell proliferation rates in the colons of *C. difficile-*inoculated pigs. Number of PCNA positive cells in jejunal **(Top)** and colon **(Bottom)** tissue. Significance of differences between treatments was determined using Mann-Whitney tests.

At the end of the experiment, PCNA staining of colon tissue demonstrated significant differences in cell proliferation rates between Clin *C. diff* and Cip *C. diff* groups and the control group (Mann-Whitney *U*-test, *p* = 0.0159 and 0.0556, respectively, Figure 2), but not between Clin and Cip groups and the Control group, indicating that this was a *C. difficile*-specific outcome (Figure 2). No differences between groups could be observed for the number of macrophages and CD3^+^ cells in colon sections or *C. difficile-*associated cell proliferation crypte/villus ratios and the number of macrophages and CD3^+^ in jejunal tissue (data not shown).

### Effects of Antibiotic Treatments on the Fecal Microbiome

We performed 16S rRNA gene amplicon sequencing and quantification of *C. difficile*-specific genes on the same fecal samples used for gene quantification in order to identify whether shifts in bacterial community structures induced by Clindamycin and Ciprofloxacin treatments increased the colonization success of *C. difficile*. In the absence of antibiotics and throughout the experiment, the fecal microbiomes contained an average of 237 ± 99 different SVs. The communities were heavily dominated by the Firmicutes (71.2±7.8% of the community on average) and Bacteroidetes (23.2±8.1% of the community on average). The dominant genera were *Prevotella* (17.1±7.6%), *Blautia* (6.6±2.9%), *Roseburia* (5.7±4.6%), a *Ruminococcaceae* SV (7.7±3.0%), and a *Lachnospiraceae* SV (5.0±2.6%).

**Figure 3:**
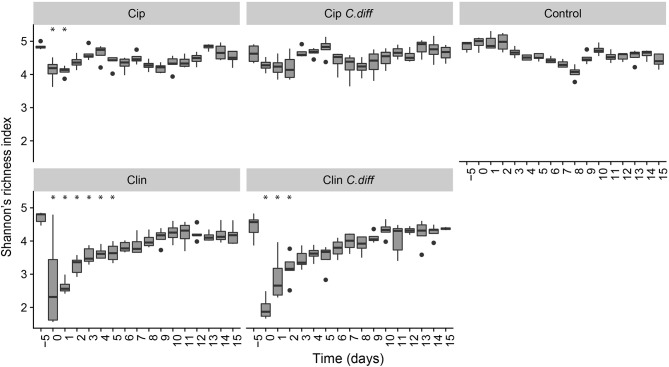
Recovery of bacterial diversity following antibiotic administration. Diversity is displayed as Shannon's H. For each treatment, asterisks represent time points which were significantly different from day −5 microbiomes (pairwise comparisons of a one way ANOVA using Tukeys's HSD, *p* < 0.01). Error bars represent standard error.

**Figure 4:**
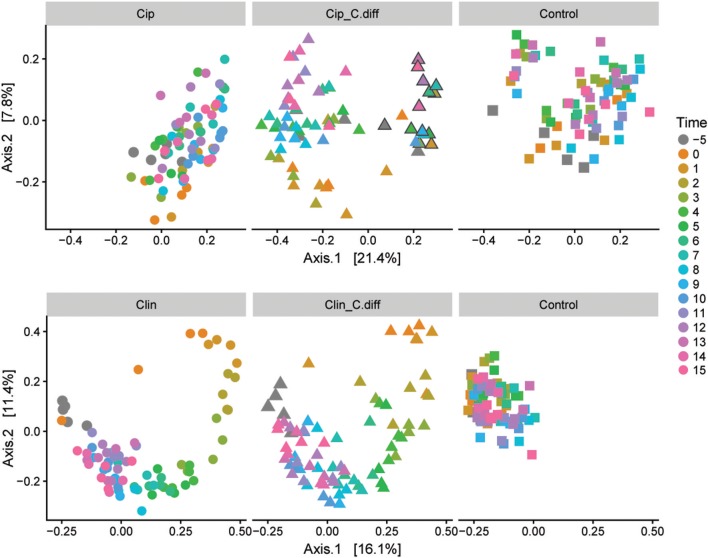
Microbial community responses to treatments. Separate Bray-Curtis PCoA ordinations of microbial community dynamics of Ciprofloxacin **(Top)** and Clindamycin **(Bottom)** treated pigs over time. Each ordination is faceted by treatment: antibiotic (left), antibiotic+ *C.difficile* (middle), and control (right). Gray outlines in the Cip. *C.diff* treatment surround samples from a single animal which exhibited a differing response.

We measured α-diversity in terms of the Shannon richness index. In the control microbiomes, α-diversity showed no clear patterns, but fluctuated significantly over time (*p* < 0.001, ANOVA, Figure 3), possibly reflecting the ongoing growth of the pigs or their adaptation to the experimental environment, or intrinsic variability. Similarly, a PCoA showed a weak, but not significant, effect of time on the community structures of Control samples (PERMANOVA, pseudo *F* = 1.16, *R*^2^ = 0.24, *p* = 0.085, Figure 4).

Ciprofloxacin treatment (Cip) resulted in significantly lower relative abundances of Proteobacteria, Bacteroidetes, Actinobacteria, Actinobacteria taxa, and unclassified Bacteria; and relative increases in Firmicutes taxa (*p* < 0.05, Welch's *t*-test). Notably, in response to treatment, members of the *Blautia* genus increased from 9.05% on day −5 to 25.2% of the community on day 0, on average (*p* = 0.07, Welch's *t*-test). Following Cip treatment on day 0, richness fell to 85.15 ± 7.62% of day −5 values and remained significantly lower than day −5 samples until day 1 (*p* < 0.01, ANOVA, pairwise comparisons; Figure 3). By day 2, richness recovered to day −5 levels. The community structure exhibited strong temporal dynamics following Cip treatment, resulting in an altered community structure (PERMANOVA, pseudo-F = 2.04, *R*^2^ = 0.36, *p* < 0.001, Figure 4). Notably, Cip treated communities were not more variable than control communities (*p* > 0.05, Tukey's HSD pairwise test on Betadisper, Figure S3).

Clindamycin treatment (Clin) resulted in a high variability between Clin samples on day 0, and only the reduction in unclassified Bacterial taxa was significantly different (*p* = 0.01, Welch's *t*-test), but the relative reduction of Firmicutes was observed, as well as the relative increase in Bacteroidetes and Proteobacteria. Within Proteobacteria, members of the *Escherichia/Shigella* genus increased from 0.1% of the community on day −5 to 35.97% on day 0, on average. α-diversity in clindamycin-treated samples remained significantly below that of untreated samples until day 5 (*p* < 0.05, ANOVA; Figure 3). The recovering community exhibited clear temporal dynamics, gradually shifting its composition over time (PERMANOVA, pseudo-F = 4.01, *R*^2^ = 0.52, *p* < 0.001, Figure 4). Relative to control communities, samples from the Clin treatment were more variable (*p* < 0.001, Tukey's HSD pairwise test on homogeneity of dispersions; Figure 4, Figure S3).

### Effects of *C. difficile* Inoculation on the Fecal Microbiome

**Figure 5:**
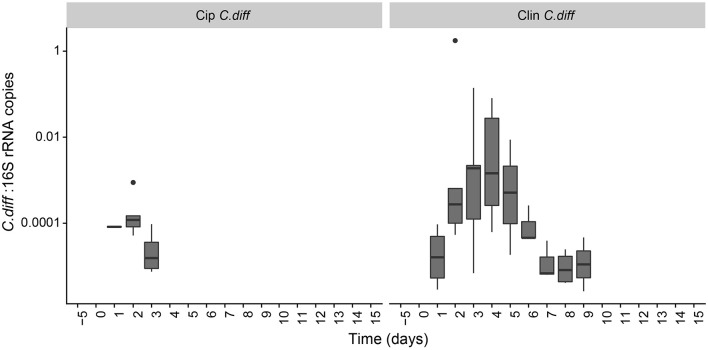
Persistence of C*. difficile* in the pig microbiome depended on the antibiotic pre-treatment. Ratio of *C. difficile-*specific 16S rRNA copies to total bacterial 16S rRNA copies in *C. difficile* treatments. Samples from other treatment groups had no detectable levels of *C. difficile-*specific 16S rRNA.

The proportion of *C. difficile* in the bacterial community, as measured by qPCR, increased over time in the fecal samples of *C. difficile-*inoculated pigs, and was undetectable in the Control, Cip, or Clin treatments (Figure 5). The temporal pattern of this surge in *C. difficile* was dependent on the antibiotic used: fecal shedding of *C. difficile* in members of the Cip *C. diff* group was detectable on days 2 and 3, peaking on day 2 (0.026 ± 0.035% of the bacterial community on average). One animal also exhibited high levels of *C. difficile* on day 1. Fecal shedding of *C. difficile* in members of the Clin *C. diff* group was higher and more variable between animals, and was detected in all animals between days 1 and 9, peaking on days 3 and 4 (2.9 ± 6.2% and 2.2 ± 3.5% of the bacteria on average, respectively). A single *C. difficile* SV from the amplicon sequencing data was highly correlated with the qPCR data (Clostridium XI, *r* = 0.90, *p* = 0; species identity confirmed on BLAST).

*C. difficile* inoculation resulted in a significant shift in both Clin *C. diff* and Cip *C. diff* microbiomes relative to the Clin and Cip groups respectively (PERMANOVA, pseudo-F = 15.04, *R*^2^ = 0.12, *p* < 0.001 and pseudo-F = 18.55, *R*^2^ = 0.11, *p* < 0.001, respectively; Figure 4). However, while time was the most discriminating factor in a PCoA including Clin, Clin *C. diff* and Control samples, *C. difficile* inoculation was the main discriminating factor for the PCoA including Cip, Cip *C. diff* and Control samples (with the exception of one animal), suggesting that the *C. difficile* inoculation had a larger impact on the fecal microbiomes of Ciprofloxacin-treated pigs relative to the antibiotic application than samples from Clindamycin-treated pigs. Notably, inoculation with *C. difficile* resulted in significantly higher community dissimilarity between samples from the same Group x Time combination than in control Clin, or Cip treatments (*p* < 0.001, TukeyHSD comparison of Bray-Curtis distances between groups; Figure S3).

**Figure 6:**
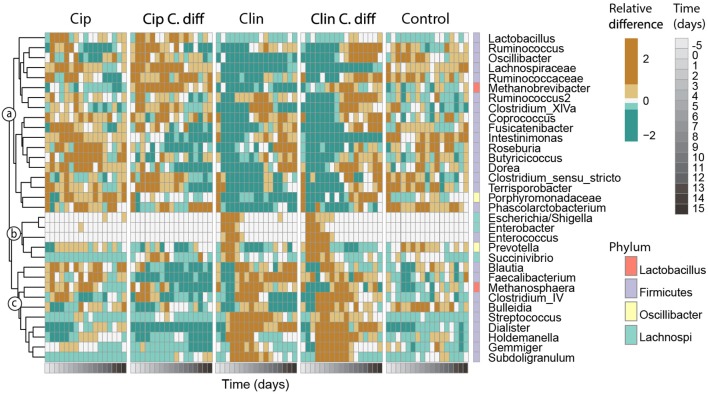
Responses of key taxa to antibiotic and *C. difficile* treatments. Thirty-three genera which exhibited high species scores in a principal response curve **(bottom)** were selected. Abundances were averaged per time point for each treatment, and centered and scaled prior to plotting. The phylum membership of each taxon is displayed on the right column, and the lowest classification of each taxon is shown on the right. Taxa were clustered into groups a–c according to their temporal response pattern using Ward's clustering method.

A principal response curve (PRC) was used to compare the effects of each treatment on the fecal microbiomes over time (Figure 6). For the whole experiment, time explained 13.6% of the variance, and group explained 35.1% of the variance. Clindamycin treatment had an overwhelming effect on community composition: relative to controls, samples from the Clin and Clin *C.diff* groups exhibited much more drastic changes in community composition than the Cip and Cip *C.dif*.

Genera with species scores greater than |1| were expected to have a larger impact on the observed community dynamics, and were selected for further investigation (Figure 6). These 33 genera represented 55.1 ± 12.1% of the community, and belonged to the Firmicutes (26 genera), Proteobacteria (3 genera), Bacteroidetes (2 genera) phyla, and 2 were Archaea. According to their patterns of abundance over time, these taxa clustered into three groups (a–c, Figure 6). Taxa in cluster *a* were more heavily affected by exposure to Clindamycin than to Ciprofloxacin within the first 5 days of recovery, increasing in relative abundance by day 8 and remaining high for the rest of the experiment. Within cluster *a* and among Ciprofloxacin-treated samples, those inoculated with *C. difficile* had a lower relative abundance of *Intestinimonas, Roseburia, Lactobacillus*, and *Butyricicoccus;* and a higher relative abundance of *Ruminococcus, Oscillibacter, Lachnospiraceae* sp., and *Methanobrevibacter* sp. (cluster a, *p* < 0.01 for all pairwise Cip:Cip *C.diff* comparisons of ANOVA). For Clindamycin-treated samples, those inoculated with *C. difficile* had significantly higher relative abundances of *Dorea, Methanobrevibacter*, and *Ruminococcaceae* sp.

Cluster *b* included taxa which were mostly absent in Control samples and became dominant in Clindamycin-treated samples between days 0 and 5. In *C. difficile* inoculated samples, this pattern was more pronounced: on day 0, *Escherichia/Shigella* exhibited a relative abundance of 42.1 ± 26.9 and 35.7 ± 27.2%, and on day 2, *Enterococcus* achieved a relative abundance of 4.0 ± 2 and 2.3 ± 0.4% in Clin *C.diff* and Clin samples, respectively (cluster b, Figure 6).

Cluster *c* consisted of taxa which became transiently abundant in Clindamycin-treated samples between days 1 and 8. Relative to samples from the Clin treatment, samples from the Clin *C.diff* treatment exhibited higher abundances of *Bulleidia* (*p* < 0.01 for all pairwise Clin:Clin *C.diff* comparisons of ANOVA).

Within the *C. difficile*-inoculated treatments, the abundance of *C. difficile* was positively correlated (pearson correlation, *r* > 0.43, *p* < 0.01) with the relative abundances of *Ruminococcus, Lachnospiraceae* sp., *Roseburia*, and *Porphyromonadaceae* sp., in the Cip *C.diff* treatment, and with *Oscillibacter, Intestinimonas, Enterococcus, Gemmiger*, and *Subdoligranulum* in the Clin *C.diff* treatment. *Ruminococcaceae* sp., *Butyricicoccus, Prevotella, Blautia, Faecalibacterium*, and *Streptococcus* were positively correlated with both treatments (pearson correlation, *r* > 0.21, *p* < 0.01).

## Discussion

Multiple studies have shown that an altered intestinal microbial community structure may allow the germination and colonization of indigenous or externally-derived *C. difficile*, which results in CDI, although the specific mechanisms behind this phenomenon are unknown (Britton and Young, 2014). In order to further the study of the relationship between CDI and native microbiota, the gut microbiome should be monitored from its pre-infection state through its infected state, which cannot be done in humans, as it requires stimulating a controlled infection. We therefore examined the colonization success of *C. difficile* in 8–10 week-old pigs following Clindamycin or Ciprofloxacin treatment.

We inoculated the pigs with strain *C. difficile* 078. In humans, this strain is regarded as mildly pathogenic, and the clinical manifestations of CDI ranges from no symptoms to severe colitis. In particular, *C. difficile* ribotype 078 toxinotype V has been isolated from affected humans as well as diarreal pigs (Goorhuis et al., 2008; Debast et al., 2009). Fecal shedding of *C. difficile* cells began 24 h after inoculation, and lasted up to 3 days in the Cip. *C. diff* treatment and up to 9 days in the Clin *C. diff* treatment, in agreement with previous findings for murine models (Reeves et al., 2011). It is possible that at the time of peak *C. difficile* shedding, histopathological changes would have been more pronounced; unfortunately no histopathological samples were taken at this time.

We did not test the effect of *C. difficile* inoculation in the absence of antibiotics. The association of CDI with prior exposure to antibiotics has been extensively studied over several decades [starting with (Bartlett et al., 1977)], consistently showing that an altered gut microbiome (i.e., through disease, aging, medication, or medical procedures) is a pre-condition for CDI (Britton and Young, 2012; Samarkos et al., 2018). In light of these findings, the omission of this experimental group is common in studies which employ animal models, as the sacrifice of additional animals is hard to justify given the available research (Reeves et al., 2011; Buffie et al., 2012; Schubert et al., 2015). Nevertheless, we do acknowledge that further study of the effect of *C. difficile* inoculation in the absence of antibiotics may aid in the understanding of the role of microbial interactions in the success of *C. difficile*.

We selected the antibiotics Ciprofloxacin and Clindamycin because Ciprofloxacin is one of the most common antibiotics used for humans worldwide, and Clindamycin has been reported to induce CDI (Britton and Young, 2014). Increased susceptibility to CDI due to Clindamycin and Ciprofloxacin administration has been reported (Best et al., 2012), although a study comparing the effects of several antibiotics in susceptibility to CDI in mice found that Clindamycin increased the risk of CDI, while no effect was detected for Ciprofloxacin-treated mice (Schubert et al., 2015). Our findings largely align with those of Schubert and colleagues. In our study, Ciprofloxacin-treated pigs were not significantly different from control pigs in terms of body temperature, and the microbiomes of Ciprofloxacin-treated pigs returned to Control diversity levels within 2 days after antibiotic administration. This is likely a result of the antibiotic's target populations: Ciprofloxacin-treated pigs exhibited relative decreases in Actinobacteria, Bacteroidetes and Proteobacterial phyla in their fecal microbiomes, and relative increases in the Firmicutes phylum, particularly the *Blautia* genus. Increases in the relative abundance of *Blautia* in response to Ciprofloxacin have been previously reported (Stewardson et al., 2015). Despite the increase in body temperature and the decrease in colon cell proliferation rates in the Cip *C. diff* group, the rapid recovery of α-diversity combined with the lack of major community dynamics within 2 days after antibiotic treatment suggests that the treatment was not strong or prolonged enough to cause major changes in the pig gut microbiome.

In contrast, Clindamycin treatment had a much more severe effect on the pigs and their fecal microbiomes. The fecal microbiomes of members of both Clin and Clin *C. diff* remained distinct from controls for 5 days after antibiotic administration, and while community composition for these treatments exhibited a trend toward recovery, it did not recover within the period studied. Body temperature was significantly higher than in controls for Clin and Clin *C. diff*, suggesting this was an effect of the antibiotic, and not of *C. difficile* administration. Notably, the relative abundance of Proteobacteria increased 1 day after disturbance, particularly *Escherichia/Shigella, Enterobacter, Enterococcus, Prevotella*, and *Succinivibrio* (members of cluster *b*) for both treatments. These taxa are rapid-growing opportunists (Fierer et al., 2007), and their dominance suggests the availability of resources following the antibiotic treatment. The increase in the relative abundance of *Blautia, Bulleidia, Holdemanella*, and other members of cluster *c* on day 2 occurred as members of cluster *b* decreased, suggesting that the latter were displaced.

Community dynamics in the fecal microbiomes of Clindamycin-treated pigs were similar regardless of whether *C. difficile* was present. For example, the *Escherichia/Shigella* genus, which is often positively correlated to *C. difficile* colonization (Schubert et al., 2015; Theriot and Young, 2015), increased in abundance in our experiment following Clindamycin treatment, even in the absence of *C. difficile* inoculation. Furthermore, while we did not find significant correlations between specific clusters of taxa and *C. difficile* abundances, the timing of growth of cluster *a* in the Clin and Clin *C.diff* treatments coincided with the end of *C. difficile* shedding in the Clin. *C. diff* treatment.

The community dynamics observed in both Clindamycin-treated groups—in particular displacement of taxa over time—are consistent with the patterns of secondary succession in microbial communities (Jurburg et al., 2017). Secondary succession, the process of recovery of disturbed communities, has been shown in a wide variety of microbial communities, from soil to gut microbiomes (Shade et al., 2012). We suggest that the competitive dynamics which arise following antibiotic treatment in the microbiome facilitate *C. difficile* establishment. This is consistent with a previous study, which showed that the time between antibiotic administration and *C. difficile* inoculation had a strong effect on the incidence of CDI (Schubert et al., 2014). The temporal sampling patterns of CDI-related microbiome research are generally relative to the patient (i.e., months), not the microbiome (i.e., days), however. Future research may consider treatments which accelerate successional dynamics, thereby reducing the window during which a *C. difficile* may become dominant.

Our finding that *C. difficile* abundances correlate positively with *Gemminger, Roseburia, Subdoligranulum, Blautia, Prevotella, Intestinimonas, Ruminococcaceae* sp., and *Enterococcus* is in agreement with previous findings, both in murine models and in human samples (Skraban et al., 2013; Schubert et al., 2015; Theriot and Young, 2015), indicating that our pig model is suitable for the study of CDI infections in humans. This experiment further provides evidence that weaned pigs can be colonized by the human pathogenic *C. difficile* strain 078, after disruption of the normal microbial community structure by pre-treatment with antibiotics. Since the post-weaning gut development and nutritional requirements of pigs closely resemble those of humans in many aspects (Litten-Brown et al., 2010; Meurens et al., 2012; Heinritz et al., 2013) antibiotic-treated pigs may be a preferred model to the currently used murine models to study the effect of nutritional-based intervention on the prevention of *C. difficile* infections in humans. Furthermore, since the gastrointestinal tract of pigs shows substantial similarities to that of humans and the pig is very similar to humans in terms of anatomy, genetics and physiology, the model described here offers an attractive intermediate for pre-clinical testing of preventive and/or therapeutic interventions for human CDI.

## Data Availability

Data is available in the NCBI SRA under accession number PRJNA528235.

## Ethics Statement

All procedures were approved by the animal experimentation board at Wageningen University & Research Center (accession number AVD401002015141) and carried out according to the guidelines of the European Council Directive 86/609/EEC dated November, 1986.

## Author Contributions

SJ and JC performed sequence and statistical analyses and wrote the manuscript. JR, MS, and JC planned and performed the experiment. PdB performed molecular analyses and sequencing. All authors contributed substantially to revisions.

### Conflict of Interest Statement

The authors declare that the research was conducted in the absence of any commercial or financial relationships that could be construed as a potential conflict of interest.
