# Supplementary material for: Successional Dynamics in the Gut Microbiome Determine the Success of Clostridium difficile Infection in Adult Pig Models
Source: Front Cell Infect Microbiol. 2019 Aug 6;9:271. doi: 10.3389/fcimb.2019.00271 (PMC6691177; doi:10.3389/fcimb.2019.00271)
Supplement: Supplementary file 1 [file Data_Sheet_1.docx]

SUPPLEMENTARY MATERIALS


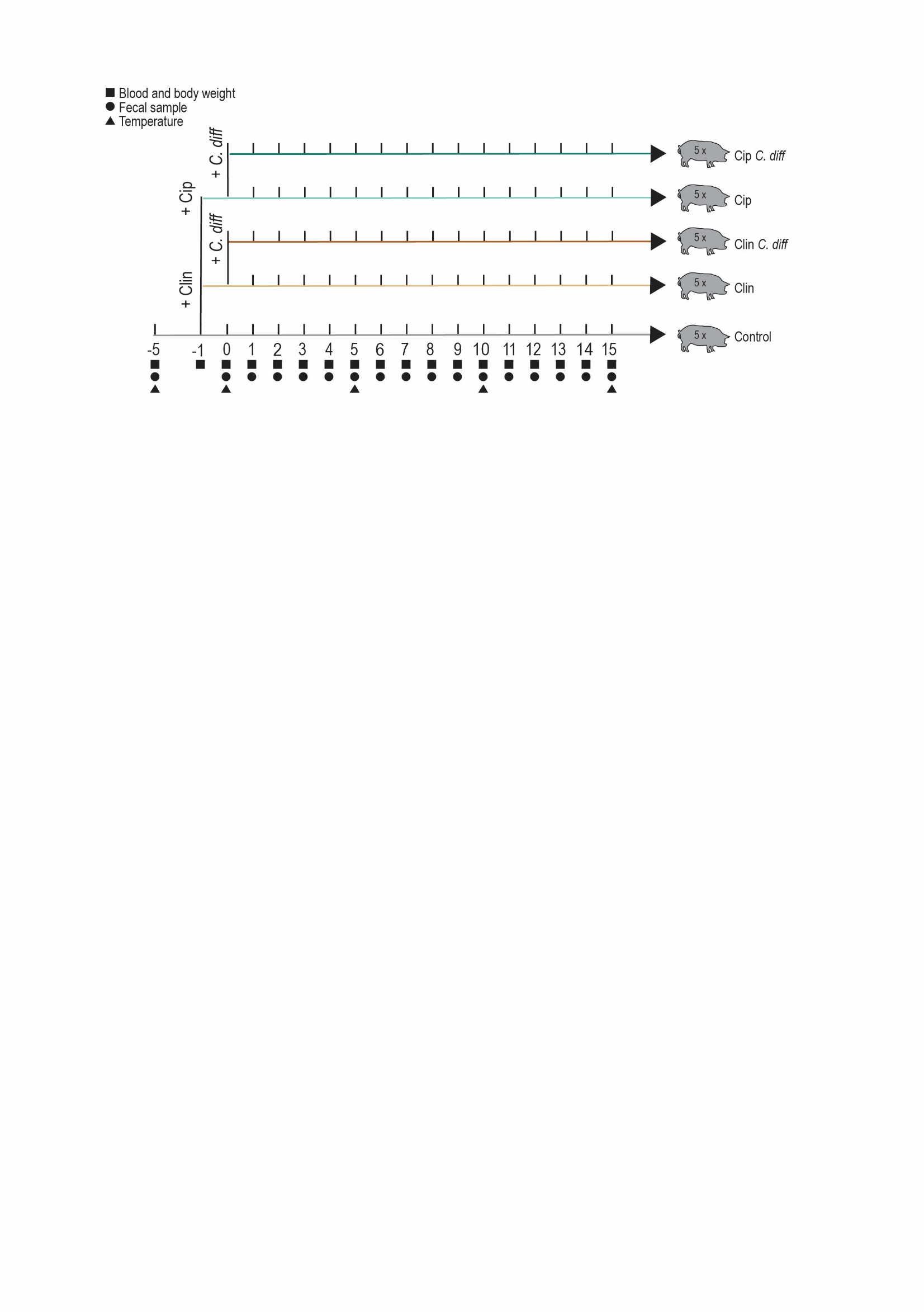


**Figure S1.** Experimental setup

**Section S2.** Supplementary materials and methods

qPCR reactions were prepared as follows. For the *C. difficile* qPCR, 45 cycles were used; for the 16S rRNA qPCR, 40 cycles were used.

| **qPCR reaction mix** |  |  | | |
| --- | --- | --- | --- | --- |
| **Component** | **Volume** |  |  |  |
| 2X Diagenode Master Mix | 12.5 µL |  | | |
| 0.2 uM reverse primer | 1.0 µL |  | | |
| 0.2 uM forward primer | 1.0 µL |  | | |
| 0.1 uM VIC probe | 1.0 µL |  | | |
| Rnase/Dnase free water | 4.5 µL |  | | |
| DNA template (5X diluted from stock) | 5.0 µL |  | | |
| Total reaction volume | 25 µL |  | | |
|  |  |  |  |  |
|  |  |  |  |  |
| **qPCR conditions** |  |  |  |  |
| **Stage** | **Repetitions** | **Temperature** | **Time (min)** |  |
| 1 | 1 | 50 °C | 2:00 |  |
| 2 | 1 | 95 °C | 10:00 |  |
| 3 | 45/40* | 95 °C | 0:15 |  |
|  |  | 60 °C | 1:00 |  |

*45 cycles were used for the *C. difficile* qPCR, and 40 cycles were used for the 16S rRNA qPCR.


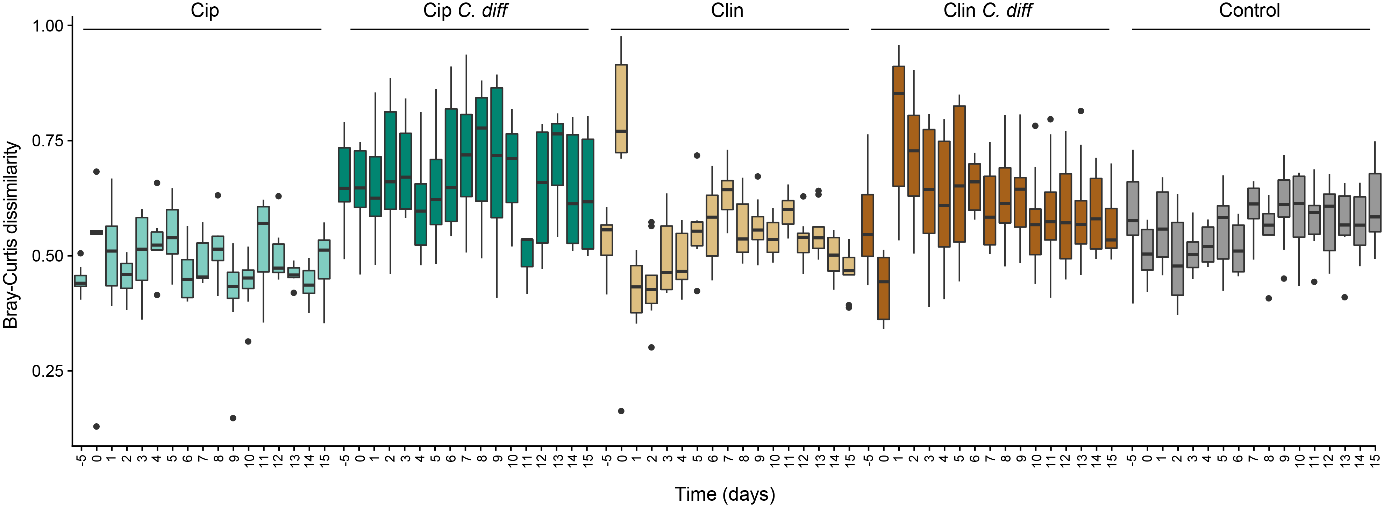


**Figure S3.** Bray-curtis distances within replicates over time
